# Mutational Analysis Reveals Functional Roles of METTL16 Domains and Residues

**DOI:** 10.3390/biology14091145

**Published:** 2025-08-29

**Authors:** Kurtis Breger, Ian P. Schowe, Noah A. Springer, Nathan J. O’Leary, Agnieszka Ruszkowska, Carlos Resende, Jessica A. Brown

**Affiliations:** Department of Chemistry and Biochemistry, University of Notre Dame, Notre Dame, IN 46556, USANSpringer@scripps.edu (N.A.S.); nathanolear@gmail.com (N.J.O.); grunia85@gmail.com (A.R.); carlos.resende@ua.pt (C.R.)

**Keywords:** methyltransferase, METTL16, *N*^6^-methyladenosine, U6 snRNA

## Abstract

Human methyltransferase-like protein 16 (METTL16) installs *N*^6^-methyladenosine on U6 small nuclear RNA (snRNA) and other RNAs. Multiple X-ray crystal structures of METTL16 have been published; however, we do not yet fully understand the structure–function relationships of specific residues. We designed 38 mutants, including seven cancer-associated mutants, and used electrophoretic mobility shift assays and single-turnover kinetic assays to better understand the functional roles of specific domains and amino acid residues in binding to U6 snRNA, formation of the METTL16•U6 snRNA•*S*-adenosylmethionine (SAM) complex, and the rate of methylation. While point mutations in the methyltransferase domain mildly weaken the binding of METTL16 to U6 snRNA, the C-terminal vertebrate conserved regions (VCRs), particularly the arginine-rich region (R382 to R388), mediate cooperative binding and contribute more to RNA binding. All METTL16 K-loop mutants displayed tighter SAM binding, suggesting that the K-loop blocks SAM binding. In addition, residues E133 and F227 are critical for stabilizing SAM binding. Mutations in the _184_NPPF_187_ catalytic core and R282A abolished methyltransferase activity. Two METTL16 somatic cancer-associated mutants (G110C and R241Dfs*2) displayed reduced methylation activity. This mutational analysis expands our understanding of how specific domains and residues contribute to substrate-binding activity and methylation of U6 snRNA catalyzed by METTL16.

## Simple Summary

RNA is more than a string of four letters (A, C, G, U) due to chemical modifications. This study investigated one human protein that chemically modifies a human RNA. We discovered certain parts of this protein that contribute to its ability to bind to the RNA and to chemically modify it. These results reveal how cancer-associated mutations alter the activity of METTL16, aiding identification of therapeutically relevant sites.

## 1. Introduction

**Figure 1:**
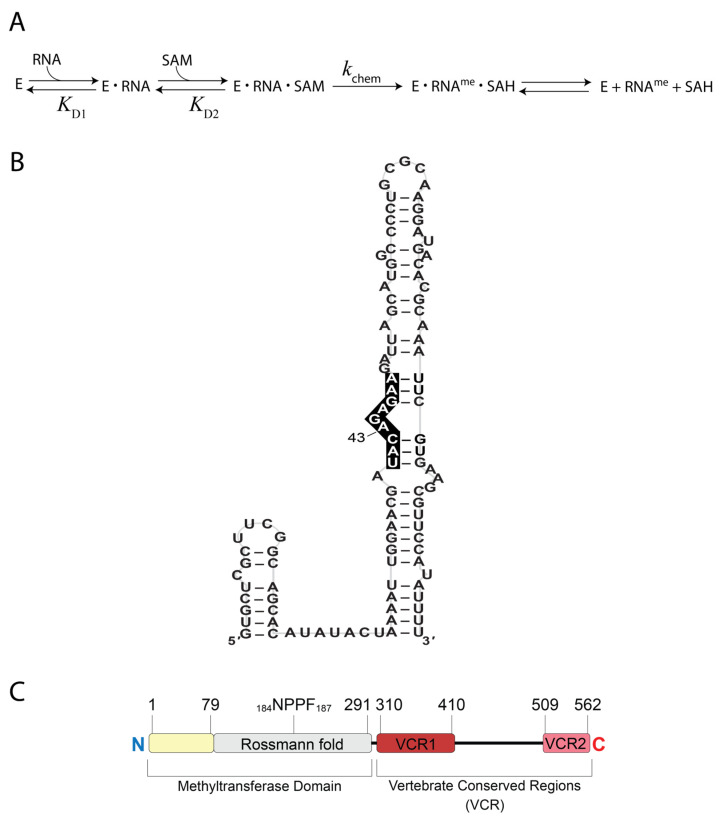
Known features of human METTL16. (**A**) The kinetic scheme of METTL16 (E for enzyme) with U6 snRNA and SAM as substrates. (**B**) Schematic of U6 snRNA with the sequence corresponding to the conserved nonamer motif (i.e., URYARDRRD) denoted by white text on a black background. (**C**) Domain organization of full-length METTL16. The methyltransferase domain contains an RNA-binding region (yellow) and a Rossmann fold (gray). The VCR contains VCR1 (dark red) and VCR2 (pink) separated by a region (residues 402–498) predicted to be disordered by the MobiDB server [22].

In humans, *N*^6^-methyladenosine (m^6^A) is an important RNA modification because it is involved in various cellular functions such as splicing [1,2], nuclear export [3,4,5], and translation [6,7,8]. The vast majority of m^6^A marks occurring in mRNAs and other RNA polymerase II transcripts are installed by the *S*-adenosylmethionine (SAM)-dependent methyltransferase-like protein 3 and 14 (METTL3 and METTL14) complex [9,10]. Notable exceptions are installed by methyltransferase-like protein 16 (METTL16) and include six hairpins within the 3′-UTR of methionine adenosyltransferase 2A (MAT2A) mRNA as well as U6 small nuclear RNA (snRNA) and its pseudogenes [10,11]. These identified substrates possess the consensus sequence URYARDRRD (A is m^6^A target; R = A or G; Y = C or U; D = A, G or U), typically located within a hairpin, bulge, or flanked by structured RNA [10,11,12,13]. The kinetic mechanism (Figure 1A) has been examined for METTL16 methylating A43 of U6 snRNA (Figure 1B) [14,15,16]. METTL16 first forms a binary complex with the U6 snRNA (METTL16•U6 snRNA) before binding SAM to form a ternary complex (METTL16•U6 snRNA•SAM) where methylation of A43 can proceed [14]. In contrast, hairpin (hp) 1 of the MAT2A mRNA reportedly proceeds by a random-order binding mechanism [16]. The methylation status of the MAT2A hairpins (hps) and U6 snRNA are biologically significant in human cells. METTL16 and MAT2A mRNA function in a feedback loop that regulates SAM homeostasis [10,11,17,18,19]. In a catalytically active spliceosome, the m^6^A43 mark of U6 snRNA resides within the UACAGA box that base pairs with the 5′-splice site such that the resulting m^6^A43-A interaction prevents exon skipping and intron retention [20].

METTL16 is organized into two regions: the N-terminal methyltransferase domain and the C-terminal region containing two vertebrate conserved regions (VCRs) separated by a disordered region (Figure 1C). The methyltransferase domain contains a Rossmann fold, a super-secondary structure that is conserved among the five known human m^6^A RNA methyltransferases. In addition, there is an RNA-binding region from residues 1 to 79 that contribute to RNA binding, primarily because residues K5, R10, R12, K14, and K16 create a highly positively charged groove that aids in binding RNA [12,21]. When these five residues were each replaced with alanine in the METTL16 core (i.e., residues 1–291), the mutant failed to methylate the MAT2A hp1 [12]. Based upon structural and mutational analyses, K47 and R279 contribute to the RNA-binding activity of METTL16 [12,17]. Other residues within the Rossmann fold, such as R82 and R282, bind to the RNA near the consensus motif based on an X-ray crystal structure of METTL16 (i.e., residues 1–310) bound to a minimized MAT2A hp1 RNA (Protein Data Bank identifier (PDB ID): 6DU4) [17]. In addition, METTL16 mutants R82A, R82E and R282E eliminated methylation activity on the MAT2A hp1 RNA [12,17]. The VCRs in the C-terminal portion of METTL16 greatly enhance binding and methylation of U6 snRNA, in addition to binding non-substrate RNAs such as the MALAT1 triple helix, but not methylation of the MAT2A hps [14,15,21]. In particular, an arginine-rich region (R382 to R388) is crucial for METTL16 methylating U6 snRNA (Figure 1B,C) [15].

In this work, we probed select amino acids of METTL16 for their substrate-binding and catalytic activities in the presence of U6 snRNA. In general, the C-terminal region is more crucial for binding to U6 snRNA than the N-terminal region. Most mutations in the methyltransferase domain of METTL16 weakened RNA-binding activity by up to ~3-fold, but METTL16 with perturbations to the arginine-rich region in the C-terminus were up to 10-fold weaker. Residues that interact with the adenosine moiety of SAM, such as E133 and F227, were observed to be crucial for SAM binding. Reducing the side chain length of residues in the K-loop improved SAM binding up to 10-fold. Most mutations within the _184_NPPF_187_ motif disrupted methylation activity to an extent that only minimal or no m^6^A product was detected under single-turnover conditions. Lastly, we analyzed various somatic cancer-associated mutations of METTL16 reported in the literature. While most cancer-associated mutants (R200Q, E408K, P460L, T549A, and R552H) did not alter the activity of METTL16, other cancer-associated mutants (G110C identified in an intestinal cancer patient and R241Dfs*2 identified in a colorectal cancer patient) nearly or completely abolished methylation activity. Overall, our study provides a greater functional understanding of how specific amino acids and domains of METTL16 contribute quantitatively to substrate binding and methylation of U6 snRNA.

## 2. Materials and Methods

### 2.1. Expression and Purification of Wild Type and Mutant METTL16

pMCSG92 plasmids encoded either *METTL16_291* (1–291) with a N-terminal His_6_-tag or full-length (FL) *METTL16* (1–562) with a C-terminal His_6_-tag. Proteins were expressed and purified as previously described [14]. A pMCSG68 vector encoding a C-terminal His_6_-tag of *METTL16* Δ1-291 (292–568) was prepared using a ligation-independent method as described previously [21,23]. Most METTL16 mutants were prepared using either an in vivo assembly (IVA) cloning strategy [24] or blunt-end ligation with the parent pMCSG92 plasmid encoding full-length *METTL16* (1–562). However, METTL16 mutants K163A/M167A, Q162A/K163A/M167A, R382A, R382A/R383A/R386A/R388A, and ΔR382-R388 were prepared by Azenta Life Sciences (Burlington, MA, USA). Sequences of all mutants were confirmed using the Sanger sequencing service provided by Azenta Life Sciences.

To express recombinant human METTL16, BL21 Gold competent *E. coli* cells (Agilent, Santa Clara, CA, USA) were transformed with desired plasmids encoding *METTL16*. Purification of METTL16 and the mutants was performed as previously described [14,21]. After sonication, METTL16 was isolated using nickel affinity chromatography, dialyzed overnight with TEV protease to remove the His_6_-tag, and flowed through the nickel column a second time. The flow-through was then loaded onto a HiLoad 16/600 Superdex column (GE Healthcare, Marlborough, MA, USA) equilibrated with the optimized reaction buffer (refer to Methyltransferase Assays) using an AKTA Pure FPLC system (GE Healthcare). Proteins were centrifuged at maximum speed (15,000 rpm) in an Eppendorf centrifuge 5424R at 4 °C to remove aggregates, and concentrations were measured using a NanoDrop OneC spectrophotometer (Thermo Fisher Scientific, Waltham, MA, USA). Final protein stocks were aliquoted and stored in optimized reaction buffer at −80 °C.

### 2.2. Electrophoretic Mobility Shift Assays (EMSAs)

U6 snRNA (5′-GUGCUCGCUUCGGCAGCACAUAUACUAAAAUUGGAAC GAUACAGAGAAGAUUAGCAUGGCCCCUGCGCAAGGAUGACACGCAAAUUCG UGAAGCGUUCCAUAUUUU-3′) was prepared using homemade T7 RNA polymerase as previously described [14]. The 5′-triphosphate of the U6 snRNA was removed using calf intestinal alkaline phosphatase (Promega, Madison, WI, USA). U6 snRNA was then 5′-radiolabeled using γ-[^32^P] ATP (~7000 Ci/mmol, PerkinElmer, Waltham, MA, USA) and T4 PNK (New England Biolabs, Ipswitch, MA, USA) per the manufacturer’s protocol. Remaining γ-[^32^P] ATP was removed using a G25 microspin column (GE Healthcare). The 5′-[^32^P]-radiolabeled U6 snRNA was folded in 20 mM HEPES pH 7.5 at 20 °C, 150 mM KCl, 10 mM MgCl_2_, 5 mM TCEP, and 10% glycerol, heated at 95 °C for 3 min, snap-cooled on ice for 5 min, and then equilibrated at room temperature for at least 30 min. Increasing amounts of METTL16 (up to 2 µM) or the appropriate METTL16 mutant (up to 20 µM) was added to 2 nM 5′-[^32^P]-radiolabeled U6 snRNA and incubated at room temperature for 60 min. Samples were loaded onto a 5% native polyacrylamide gel (19:1 acrylamide/bisacrylamide, 1× Tris-borate (TB) buffer, and 1 mM MgCl_2_) and resolved at 130 V for 150 min. Gels were then wrapped in plastic wrap and exposed to a Phosphorimager screen overnight. Screens were scanned using the Fluor stage AmTyphoon installed in an Amersham Typhoon IP (GE Healthcare). ImageQuant TL (ver. 8.1) software was used to quantify bands. The fraction of RNA bound (nM) was plotted versus the concentration of protein (nM) and fitted to the Hill equation:

(1) $$y=\;\frac{R_{total\;}\times \;{P_{total}}^{n}}{{K_{D1}}^{n}\;+{{\;P}_{total}}^{n}}$$

where *R*_total_ is the total RNA concentration (nM), *P*_total_ is the total protein concentration (nM), the *K*_D1_ is the dissociation constant for the METTL16•U6 snRNA complex, and *n* is the degree of cooperativity.

### 2.3. Circular Dichroism (CD) Spectroscopy

Protein samples were diluted to 3 µM in 450 µL of CD buffer (10 mM NaPO_4_ pH 7, 150 mM KCl, 10 mM MgCl_2_, and 1 mM TCEP). All proteins were centrifuged at 15,000× *g* for 30 min at 4 °C to remove aggregates. Samples were then dialyzed overnight in 250 mL of CD buffer using a 15 kDa-MWCO Tube-O-Dialyzer (G-Biosciences, St. Louis, MO, USA). All CD spectra were recorded at 20 °C on a Jasco J815 spectrophotometer using quartz cuvettes with a 0.1 cm path length. Measurements were taken using an 8 s integration time, 20 nm/s scanning speed, 1 nm band width, and averaging 6 spectra scanning between 200 and 250 nm. A buffer spectrum was subtracted from each protein sample spectrum. Spectra were plotted as molar ellipticity (deg × cm^2^/dmol) versus wavelength.

### 2.4. Methyltransferase Assays

The U6 snRNA with the A43 position [^32^P]-radiolabeled was prepared using splint ligation as previously described [14]. The *k*_chem_ and equilibrium dissociation constant (*K*_D2_) for the METTL16•U6 snRNA•SAM complex was determined using optimized reaction buffer (20 mM HEPES pH 7.5 at 20 °C, 150 mM KCl, 10 mM MgCl_2_, and 5 mM TCEP) under the following conditions: 5 µM METTL16 mutant was pre-equilibrated with 0.5 µM U6 snRNA and time courses were initiated upon addition of SAM (Cayman Chemical Item No. 13956) at 10, 50, 100, 250, 500, and 1000 µM. At each time point (1–30 min), a 10 µL aliquot was quenched using 100 µL of phenol-chloroform. RNA from each aliquot was digested into nucleosides and spotted onto TLC plates to separate radiolabeled [^32^P]-A43 and [^32^P]-m^6^A43 as previously described [14]. Dried TLC plates were exposed to Phosphor screens overnight and scanned using an Amersham Typhoon Phosphorimager (GE Healthcare). ImageQuant TL software (ver. 8.1) was used to quantitate the [^32^P]-A43 and [^32^P]-m^6^A43 signals. Origin 2018b software was used to fit the data of [^32^P]-m^6^A43 product concentration (nM) versus time (min) to Equation (2):

y = *A* × (1 − e^(−*k*obs×*t*)^) (2)

where A is the scaling constant, *k*_obs_ is the observed rate constant, and *t* is the time in min [25]. The *k*_obs_ constants were then plotted versus the respective SAM concentrations and fit to Equation (3):

(3) $$k_{obs}=\frac{k_{chem}\times [SAM]}{K_{D2}+[SAM]}$$

where *k*_obs_ is the observed rate constant, *k*_chem_ is the single-turnover rate constant (i.e., rate encompassing steps from METTL16 preincubated with RNA to the methyl transfer), [SAM] is the concentration of SAM, and *K*_D2_ is the apparent equilibrium dissociation constant for the METTL16•U6 snRNA•SAM complex.

No generative artificial intelligence (GenAI) has been used in this paper (e.g., to generate text, data, or graphics, or to assist in study design, data collection, analysis, or interpretation).

## 3. Results

### 3.1. Arginine-Rich Region Is Critical for METTL16 to Bind to U6 snRNA

**Figure 2:**
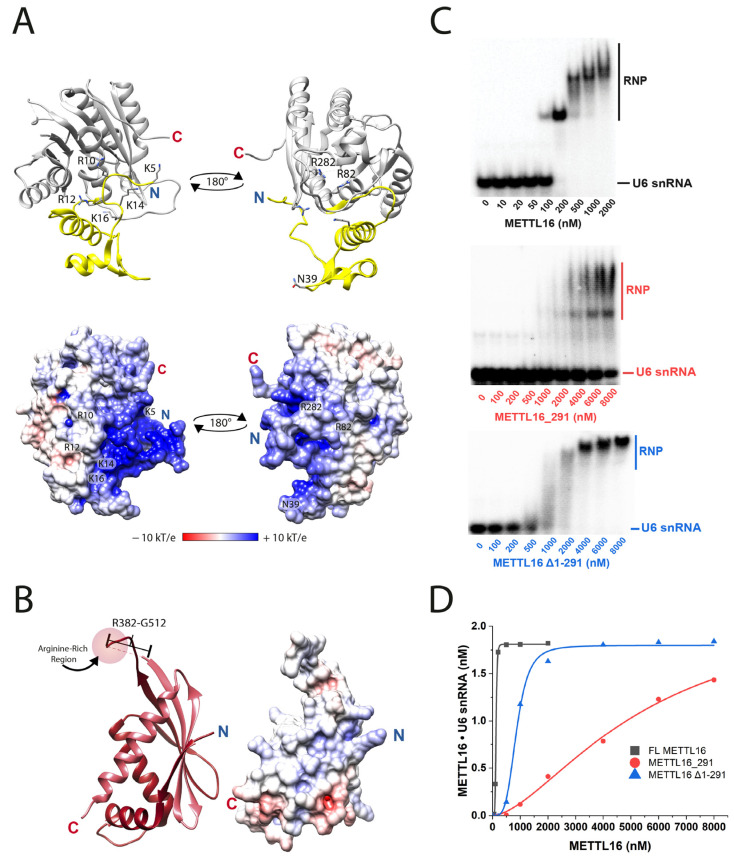
Residues throughout METTL16 are involved in U6 snRNA binding. (**A**) The methyltransferase domain possesses K5, R10, R12, K14, and K16 in an RNA-binding region and R82 and R282 in the RNA-binding groove (PDB ID: 6DU4). Surface electrostatic potential maps of the methyltransferase domain structures are below each respective orientation. (**B**) The VCR structure possesses an arginine-rich region (R382 to R388) that resides in an unsolved region (R382 to G512, shown as dashed line) between VCR1 (dark red) and VCR2 (dark pink) (PDB ID: 6M1U). The surface electrostatic potential map of the VCRs is shown to the right. (**C**) Representative images for native gel-shift assays resolving binary complex formation for full-length METTL16 (top), METTL16_291 (middle), and METTL16Δ1-291 (bottom). (**D**) Plot of binary complex formation versus concentration of full-length METTL16 (black squares), METTL16_291 (red circles), and METTL16Δ1-291 (blue triangles). This plot was created using the densitometry data determined from gel images shown in panel C. Three-dimensional structures were visualized using Chimera and electrostatic potential maps were generated using the PDB2PQR version 3.4.1 [26,27].

The first step in the kinetic pathway is for METTL16 to bind to U6 snRNA (Figure 1A). Therefore, we examined various mutants to probe which residues and regions of METTL16 contribute to the recognition of U6 snRNA. Mutants include deletions of entire domains or substitutions/deletions of residues residing in the RNA-binding region (amino acids 1–79), within the Rossmann fold, or an arginine-rich region near the C-terminal end of VCR1 (Figure 2A,B). To measure binding affinity, we first employed isothermal titration calorimetry; however, these attempts were unsuccessful because METTL16 proteins were prone to aggregation in the sample cell. Instead, electrophoretic mobility shift assays (EMSA) were employed to obtain apparent equilibrium dissociation constants (*K*_D1_) for the METTL16•U6 snRNA complexes. EMSAs were performed by adding increasing concentrations of full-length METTL16, METTL16_291 (i.e., methyltransferase domain) or METTL16Δ1-291 (i.e., C-terminal domain) to U6 snRNA. The gel images showed up to two distinct bands representing the METTL16•U6 snRNA ribonucleoprotein (RNP) complex (Figure 2C; see complete native gel-shift images in Figure S5). The binding stoichiometry is 1:1 for METTL16:U6 snRNA; therefore, these bands likely represent two conformationally distinct complexes, which is consistent with previous observations [14,15]. Quantitation of the binding affinity revealed that formation of the RNP complex is a cooperative process, for the data points did not follow simple hyperbolic or quadratic binding models and were visually sigmoidal. Therefore, *K*_D1_ values were extrapolated using the Hill equation (Equation (1)), generating values of 132, 5200, and 814 nM for full-length METTL16, METTL16_291 or METTL16Δ1-291, respectively, binding to U6 snRNA (Figure 2D and Table 1). This binding trend was consistent with previous reports showing the C-terminal domain of METTL16 is critical for the methylation of U6 snRNA [15]. Interestingly, the degree of cooperativity was 5 for full-length METTL16 but only 1.6 for the methyltransferase domain, suggesting that various regions/residues of the C-terminal domain act cooperatively to bind to U6 snRNA and further supporting the possibility of conformationally distinct RNPs (Table 1). These results suggested that the C-terminus contributes more to the binding of the U6 snRNA than the methyltransferase domain.

Our next objective was to determine which specific residues in the N- and C-terminal domains contribute to RNA-binding activity. A previous study indicated an RNA-binding region, specifically within residues 1–40 (Figure 1B and Figure 2A), is important for METTL16 binding to the MAT2A hp1 [12]. Therefore, we created a series of METTL16 mutants targeting positively charged residues in the RNA-binding region: K5A, K5A/R10A, K5A/R10A/R12A, K5A/R10A/R12A/K14A, and K5A/R10A/R12A/K14A/K16A (Figure 2A). Starting with METTL16 K5A, we observed no significant change in RNA-binding activity with U6 snRNA but each additional alanine substitution resulted in a slightly weaker *K*_D1_ until four and five substitutions resulted in an approximately 2.5-fold difference (Table 1). METTL16 N39A was also tested due to its proximity to the hp1 loop region; however, the *K*_D1_ of METTL16 N39A•U6 snRNA was similar to full-length METTL16 [17]. In the Rossmann fold, two positively charged residues (R82A and R282A) were investigated based upon solved 3D structures of METTL16 (Figure 2A) [12,17,21]. Only ~2-fold weaker binding activity was detected for these METTL16 mutants binding to U6 snRNA (Table 1). Similarly, METTL16 F187G and F187W, where F187 can pi-stack with the acceptor adenosine in the METTL16•MAT2A hp1 crystal structure (Figure S2), displayed only a ~2.3-fold increase in *K*_D1_ (Table 1). The *K*_D1_ values were also determined for other mutations to the Rossmann fold, such as the K-loop residues, SAM-binding pocket, and _184_NPPF_187_ catalytic core, but none elicited any significant changes to binding U6 snRNA (Table S1). Interestingly, the degree of cooperativity ranged from 4 to 14 for the mutants, with most of them exhibiting more cooperativity than full-length METTL16 (Table 1 and Table S1). The C-terminal domain has an arginine-rich region (Figure 2B) and all those mutants displayed weaker RNA-binding activity: 2-fold for R382A, 5-fold for R382A/R383A/R386A/R388A and 10-fold for ΔR382-R388. Notably, the quadruple and deletion mutant had cooperativity values less than 3, suggesting this region may partially contribute to the high cooperativity (Table 1). Because several of the mutants displayed relatively weak RNA-binding activity, we employed CD spectroscopy to check if the mutant protein was folded similar to full-length METTL16. None of the mutants examined, excluding those introducing large deletions to METTL16 (i.e., METTL16_291 and METTL16Δ1-291), revealed any alterations to the structural fingerprint (Figure S1A,B). Future research should also examine the thermal stability and consider structural modeling to better understand the structure–function relationships of the METTL16 mutants presented herein.

In addition to testing RNA-binding activity, the abovementioned METTL16 mutants were also subjected to single-turnover kinetic analysis to probe for any perturbations to SAM binding or catalysis. In general, most mutants yielded parameters similar to full-length METTL16 (Table S2), except for residues residing in the SAM-binding pocket (R82 and F187) or catalytic core (F187); therefore, these relevant mutants are discussed below with other site-related mutants. However, METTL16 R282A was catalytically dead (Table S2), a result that is similar to the METTL16 core R282E not methylating the MAT2A hp1 [12]. Among the METTL16 mutants examined herein, the residues that contribute the most to the RNA-binding activity of METTL16 are those in the C-terminus, particularly the arginine-rich region.

### 3.2. SAM Binding Improves with Small, Neutral Side Chains in K-Loop

**Figure 3:**
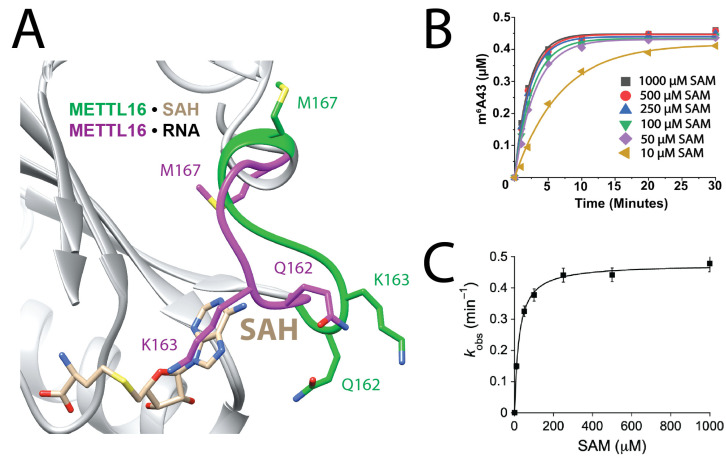
K-loop regulates SAM binding affinity. (**A**) The K-loop structure occludes SAM from entering the active site. The K-loop for the METTL16•RNA complex is indicated in magenta (PDB ID: 6DU4); the K-loop for the METTL16•SAH complex is indicated in green (PDB ID: 6B92). RNA is not visible in this view of the crystal structure. (**B**) Single-turnover kinetic analysis of 5 µM METTL16 K163A preincubated with 0.5 µM U6 snRNA was added to various concentrations of SAM (10–1000 µM). The R^2^ values ranged from 0.99416 to 0.99860; please see Supplementary File S1 for exact R^2^ values of each curve fitting. (**C**) The plot of *k*_obs_ values versus SAM concentrations, yielding a *k*_chem_ of 0.48 ± 0.01 min^−1^ and *K*_D2_ of 23 ± 2 µM. All reported error values (solid vertical lines) are from data fitting, whereby R^2^ = 0.99746 for the plot in (**C**).

The second step in the kinetic pathway is for the METTL16•U6 snRNA complex to bind SAM (Figure 1A). A structural feature unique to METTL16 is the K-loop, aptly named after K163 because it is thought to regulate SAM binding [17]. Superposition analysis shows that K163 in METTL16•MAT2A hp1 RNA crystal structure would sterically clash with *S*-adenosylhomocysteine (SAH) in the METTL16•SAH crystal structure, suggesting that K163 blocks SAM binding (Figure 3A) [17,21]. A previous study showed that METTL16 K163A had greater activity than full-length METTL16 at low SAM concentrations (1–5 µM SAM). The underlying reason for improved activity of METTL16 K163A was presumed to be tighter binding to SAM [17]. Further structural analysis suggests other K-loop residues, namely Q162 and M167, may also affect SAM binding because Q162 interacts with SAM via its peptide backbone and M167 has different orientations in the two different structural complexes (Figure 3A) [17,21]. Three single-point mutants (Table 2) were generated and their contributions to SAM binding and catalysis were analyzed using single-turnover assays, which measure *K*_D2_ of the METTL16•U6 snRNA•SAM complex and the rate of methylation, *k*_chem_ (Figure 1A and Figure 3B,C). The U6 snRNA was prepared with a 5′-[^32^P] radiolabeled phosphate at A43 (i.e., the adenosine targeted for methylation) so that unreacted [^32^P]-A43 and [^32^P]-m^6^A43 could be separated by thin layer chromatography (TLC). METTL16 mutants were in 10-fold molar excess relative to the U6 snRNA. METTL16 Q162A, K163A, and M167A had better affinity to SAM than full-length METTL16 by 1.6-, 5.5- and 2.9-fold, respectively (Figure 3B,C and Table 2). Likewise, all three METTL16 mutants maintained a rate of methylation similar to full-length METTL16. Next, we created a double (K163A/M167A) and triple (Q162A/K163A/M167A) mutant and SAM binding was ~9-fold better than full-length METTL16 (Table 2). Thus, residues within the K-loop appear to regulate SAM binding, and not catalysis, presumably via steric exclusion.

### 3.3. Stabilizing Adenosyl Moiety in Binding Pocket Is Critical to SAM Binding

**Figure 4:**
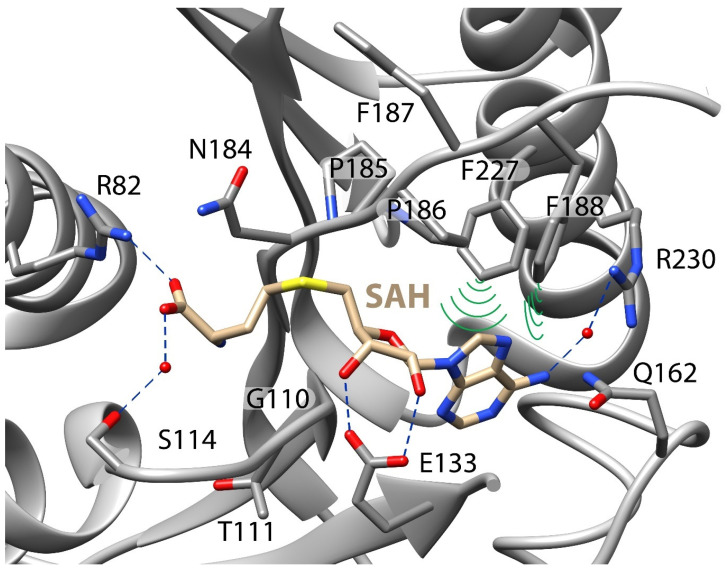
The SAH-binding pocket and catalytic core motif of METTL16. Putative SAM-binding residues are labeled as well as the catalytic core, _184_NPPF_187_ (PDB ID: 6GFK). Hydrogen bonds are indicated by blue dashed lines while green arcs represent hydrophobic interactions. R200 resides within an unsolved region of the protein; location of T216 is shown in Figure S3. PDB 6GFK was chosen because it has more residues modeled in the SAH-binding pocket than PDB 6B92. Alternative views of binding pocket are presented in the following review articles: Ruszkowska et al. [28] and Breger et al. [31].

In addition to the K-loop, an X-ray crystal structure of METTL16_291•SAH revealed a multitude of interactions that likely mediate the binding of SAM: salt bridges (R82), hydrogen bonds (E133), water-mediated hydrogen bonds (S114, R230) and hydrophobic interactions (F188, F227) (Figure 4) [21]. Eight single-point mutants (Table 3) were generated and their contributions to SAM binding and methylation of U6 snRNA were analyzed using single-turnover assays. The side chain of R82 potentially participates in a salt bridge interaction with (i) the carboxylate group of SAM as suggested by the METTL16•SAH crystal structure (PDB ID: 6B92) and/or (ii) possibly RNA given its close proximity to hp1 in the METTL16•MAT2A hp1 binary complex (PDB ID: 6DU4) (Figure 4) [12,21]. METTL16 R82A bound to SAM only 2-fold weaker but the rate of methylation decreased 48-fold (Table 3). Class I SAM methyltransferases have a conserved *GXG* motif (_110_GTG_112_ in METTL16), which partially defines the binding pocket near the methionine and adenosine moiety of SAM [28,29,30,31]. The _110_GTG_112_ motif enables water-mediated hydrogen bonds involving S114 and carboxylate group of SAH (Figure 4) [31]. The catalytic efficiency of METTL16 T111A was similar to full-length METTL16; however, the catalytic efficiency of S114A was reduced by ~3-fold, mainly due to weaker SAM binding (Table 3). The carboxyl side chain of E133 is predicted to form direct hydrogen bonds with both the 2′ and 3′ ribose hydroxyls of SAM (Figure 4). METTL16 E133A greatly destabilized SAM binding, for *K*_D2_ was greater than 1 mM and the rate constant for methylation was reduced by at least 14-fold relative to full-length METTL16 (Table 3). Two hydrophobic interactions are predicted to stabilize the adenosine moiety of SAM: F188 and F227 (Figure 4). Surprisingly, the residues had different trends. METTL16 F188A exhibited a 3-fold increase in the *K*_D2_ and a roughly 31-fold decrease in the *k*_chem_ value (Table 3). In contrast, METTL16 F227A had a drastic shift in the *K*_D2_ value to >1 mM but a mild 1.6-fold drop in *k*_chem_ compared to full-length METTL16 (Table 3). This result suggested that F227 participates primarily in SAM binding whereas F188 aids catalysis. When the METTL16•MAT2A hp1 structure is superposed with METTL16•SAH structure, the T216 side chain of the RNA-bound complex is located optimally for forming a hydrogen bond with the O2′ in the ribose of SAM (Figure S3) [17,28]. METTL16 T216A exhibited a slower *k*_chem_ of 0.29 min^−1^ but a *K*_D2_ that was 3-fold less than full-length METTL16. METTL16 R230 appears to engage in a water-mediated hydrogen bond with *N*^6^ of the adenosyl moiety (Figure 4); however, METTL16 R230A had a ~2-fold tighter *K*_D2_ than full-length METTL16, suggesting that the water-mediated hydrogen bond, if it exists, mildly impedes SAM binding (Table 3). Please note that all the mutants (E133A, F188A, F227A) with extreme decreases in activity maintained a CD spectrum similar to full-length METTL16, suggesting the mutations did not significantly alter protein secondary structure (Figure S1D). From testing residues in the SAM-binding pocket, we found that non-covalent interactions stabilizing the adenosyl moiety of SAM enhanced binding the most but not as much as the K-loop.

### 3.4. Mutations to Catalytic Core Greatly Reduce Activity of METTL16

The Rossmann folds of Class I SAM-dependent methyltransferases have a highly conserved catalytic core, which corresponds to _184_NPPF_187_ in METTL16 (Figure 4). Therefore, we investigated single- and multiple-point catalytic core mutants under single-turnover conditions to parse out their roles in methylation. As observed previously using the MAT2A hp1 as the RNA substrate, no activity was observed for METTL16 N184A and P185A/P186A mutants when U6 snRNA is the substrate (Table 4) [11,12,17]. Even replacement of the _184_NPPF_187_ core with the catalytic core DPPW from METTL3 abolished activity of METTL16 N184D and N184D/F187W. Surprisingly, METTL16 F187G possessed a measurable *k*_chem_, albeit approximately 165-fold less than full-length METTL16, and binding to SAM was only 2-fold weaker than full-length METTL16 (Table 4). However, METTL16 F187W exhibited methyltransferase activity close to that of full-length METTL16, suggesting that the N184D mutation leads to the inactivity in the DPPW core substitution of N184D/F187W. METTL16 mutants P185A/P186A and N184D/F187W appear to maintain their folded structure because CD spectra overlay with full-length METTL16 (Figure S1E). As expected, the methylation activity of METTL16 depends on the _184_NPPF_187_ core regardless of RNA substrate.

### 3.5. Catalytic Activity of METTL16 Cancer-Associated Mutants Varies from Innocuous to Inactive

After establishing the structure–function relationships of residues that impact the activity of METTL16, we were then interested in probing the functional impact of mutations to the *METTL16* gene that have been associated with human health. Databases such as the Catalogue of Somatic Mutations in Cancer (COSMIC; https://cancer.sanger.ac.uk/ (accessed on 29 November 2021)) and the National Cancer Institute Genomic Data Commons (NCI GDC; https://gdc.cancer.gov/ (accessed on 29 November 2021)) revealed multiple mutations [32,33]. We selected seven somatic cancer-associated mutations in various regions of METTL16 to determine their potential perturbations to RNA/SAM binding and methylation activity (Figure S4A). METTL16 G110C, which was identified in intestinal cancer, did not significantly alter binding to U6 snRNA; however, the *K*_D2_ value for SAM binding was greater than 1000 µM and the observed rate constant at 1 mM SAM was barely measurable: 0.005 ± 0.003 min^−1^ (Table 5 and Figure S4B). METTL16 R200Q led to a 2-fold decrease in the *K*_D2_ for the ternary complex but no other changes to *K*_D1_ or the *k*_chem_ values were observed relative to full-length METTL16 (Table 5, Table S1 and Figure S4C). Various frameshift mutants near R241 have been identified in patients with colorectal cancer [33,34]. One example is the mutant R241Dfs*2, which is missing approximately 50 amino acids from the C-terminal end of the Rossmann fold as well as the entire C-terminal domain (Figure S4A). This truncated METTL16 mutant resulted in the weakest RNA binding affinity of all mutants examined: a *K*_D1_ of 6.61 µM (Table 1, Table 5 and Table S1). This binding is approximately 38-fold weaker than full-length METTL16 and 1.4-fold weaker than METTL16_291, which has the entire Rossmann fold. The other cancer-associated METTL16 mutants (E408K, P460L, T549A, and R552H) did not result in any changes for substrate binding or methylation activity (Table 5 and Figure S4D). Within the scope of cancer-associated mutants examined herein, an extreme truncation and even single-point mutations to METTL16 displayed altered methylation activity, suggesting that these mutants could potentially contribute to cancer-associated phenotypes through increased or decreased catalytic activity. Thus, correlative studies examining the expression levels of METTL16 need to also consider the activity level of METTL16 for a more holistic assessment.

## 4. Discussion

The cellular roles of METTL16, an essential human protein [35], are diverse, such as regulating SAM homeostasis [10,11,17,18] and ferroptosis [36,37,38,39], promoting translation [10,40,41,42,43], and disrupting MRE11-mediated DNA end resection [44], just to name a few. Despite the growing physiological relevance of METTL16, this m^6^A methyltransferase has a limited number of methylation targets, namely U6 snRNA and MAT2A hps in humans [10,11,12,18,45]. Our understanding of METTL16 methylating the MAT2A hps is comparatively greater than METTL16 methylating U6 snRNA, including structure–function analyses. Herein, we examined the activity of 38 mutants methylating U6 snRNA. Consistent with previous findings, METTL16 depends more on the C-terminal VCRs, particularly the arginine-rich region, rather than the methyltransferase domain, to form a strong interaction with U6 snRNA (Table 1) [15]. In contrast, the binding of full-length METTL16 versus only the methyltransferase domain to the MAT2A hp1 differs by only 2.6-fold [15]. Notably, the electrostatic surface potential map of the methyltransferase domain (Figure 2A) of METTL16 shows a large positively charged groove whereas the electrostatic surface potential map of the VCRs (Figure 2B) does not show any notable positively charged patches, although positively charged residues 382–512 are not in the model. For the point mutants targeting the RNA-binding activity of METTL16, we discovered that most mutants show only a mild 2-fold reduction in complex formation versus full-length METTL16, suggesting that there are multiple positively charged residues to compensate for the loss of one or a few (Table 1 and Table S1). Additionally, it is not yet known what regions of U6 snRNA are recognized by each of the two domains; that structural information may also provide insights into how the METTL16 mutants maintains relatively strong binding activity. Interestingly, METTL16 displays a striking degree of cooperativity when binding to U6 snRNA and much of this cooperativity appears to originate from the C-terminal domain or crosstalk between the two domains (Table 1 and Table S1). Multi-domain RNA-binding proteins are known to exhibit cooperative interactions and unstructured regions are sometimes involved [46,47]. We speculate that the regions of METTL16 contributing to RNA binding cooperativity include the arginine-rich region (R382 to R388), which is near the predicted disordered region (residues 402–498) [22], and possibly the positively charged residues 301–310 that link the N- and C-terminal domains (Figure 1C). RNA structural cooperativity is another possibility and that might play a role in the cooperative model that has been proposed for the methylation of U6 snRNA [15]. However, one limitation is that these binding assays use an in vitro transcribed U6 snRNA, which is not physiologically identical to the post-transcriptionally modified U6 snRNA that METTL16 is likely to encounter inside cells [48]. When METTL16 methylates A43 during the biogenesis of U6 snRNA is unknown, but it is thought to occur early in the biogenesis pathway [45,49]. Similarly, these binding assays use recombinant human METTL16 expressed in *E. coli* so there are no post-translational modifications. There is one report in the literature where phosphorylation of METTL16 at S419 inhibits binding to U6 snRNA and the MAT2A hp1 based on biotinylated RNA pulldown experiments [44]. There is also a proposal that the methylation activity of METTL16 will increase when K229 is lactylated [50]. Another consideration is how protein–protein interactions alter the activity of METTL16 because lower expression levels of the METTL16-binding partner SSB results in METTL16 binding to less MAT2A mRNA and U6 snRNA [51].

In contrast to the other human m^6^A RNA methyltransferases, METTL16 binds to SAM relatively weakly: 126 µM for METTL16 versus 1–7 µM for METTL3/14, METTL5/TRMT112, and ZCCHC4 [14,16,31,52,53]. K163 appears to be the major reason for weaker binding based upon previous X-ray crystal structures (Figure 3A) as well as our kinetic analysis (Table 2) [17] Changing the side chain of K163 to alanine led to a ~6-fold tighter binding of SAM to METTL16 K163A•U6 snRNA (Table 2) and a ~6-fold increase in methylated MAT2A hp1 catalyzed by METTL16 K163A [17]. Our kinetic results show that other nearby residues in the K-loop, Q162 and M167, also weaken SAM binding; however, replacing all three residues (i.e., Q162, K163 and M167) with alanine did not show an additive nor synergistic increase in SAM binding. Instead, binding appeared to plateau at ~13 µM (Table 2), which is closer to the values measured for other human m^6^A RNA methyltransferases and the lower limit of intracellular SAM (0.7–2 µM) that is tolerable in a METTL16 K163A cell line [10,14,16,52,53]. Residues Q162, K163 and M167 are highly conserved among chordates; however, despite encoding K163 and M167, these residues do not appear to be functionally equivalent in *Caenorhabditis elegans* [21,54]. Another interesting point for the METTL16 Q162A/K163A/M167A mutant is that the *k*_chem_ value approached 2 min^−1^, which is 3.5-fold faster than full-length METTL16 (Table 2). This result suggests that conformational changes associated with the K-loop may represent a rate-limiting step for METTL16 to methylate U6 snRNA.

The SAM-binding pocket is lined with multiple residues that facilitate various non-covalent interactions with SAM (Figure 4). Among the mutated residues examined herein, the two most critical residues appear to be E133, which forms two hydrogen bonds with the ribose hydroxyl moieties of SAM, and F227, which pi stacks with adenine of SAM (Figure 4). All catalytically active human m^6^A RNA methyltransferases have residues that can coordinate with the 2′ and 3′ hydroxyls of the SAM ribose: METTL3 has Q550, METTL16 has E133, METTL5 has D81, and ZCCHC4 has D225 [21,31,52,53,55]. While METTL3 Q550A exhibits diminished methylation activity, it only drops to roughly less than 2-fold that of wild type METTL3 because R536 and N549 in METTL3 also interact with the ribose hydroxyl groups [53]. In contrast, METTL16 appears to utilize only E133 to coordinate the hydroxyls of the ribose (excluding the possibility of T216 in the ternary complex), which may explain the 94-fold decrease in methylation activity for METTL16 E133A versus full-length (Table 3) [21]. For F227, this residue remains positioned adjacent to adenine of SAM in all X-ray crystal structures of METTL16 (Figure 4). The fixed position of F227 suggests that this residue primarily functions to aid in binding the SAM metabolite and indeed our measured *K*_D2_ is greater than 1 mM SAM for METTL16 F227A (Table 3). Interestingly, F188 is oriented towards adenine of SAM in the METTL16•SAH complex (PDB ID: 6GFK) but is oriented towards the methyl acceptor adenosine in the METTL16•MAT2A hp1 complex (PDB ID: 6DU4) [12,17]. While *K*_D1_ and *K*_D2_ of METTL16 F188A increased by 2- and 3-fold, respectively, the 500-fold drop in the rate constant compared to full-length METTL16 suggests that this residue aids in catalysis more than formation of the ternary complex (Table 3 and Table S1). Understanding the roles of specific residues in SAM binding may enable the development of SAM analogs that are specific for METTL16 so that METTL16-specific methyl marks can be tracked inside cells [56,57,58,59,60].

As shown previously by others, our mutational analysis confirmed that _184_NPPF_187_ is the catalytic core of METTL16, for METTL16 mutants N184A, N184D, P185A/P186A, and N184D/F187W did not show any measurable activity (Table 4) [11,12,17]. The catalytic core motif of METTL3 is DPPW; however, this motif is not functional in METTL16. METTL16 can tolerate F187W but aspartate is not interchangeable with asparagine, likely because of electrostatic repulsion between the carboxylate of SAM and D184 in the METTL16 mutant (Table 4 and Figure 4). This electrostatic repulsion is not a problem for METTL3 because the catalytic aspartate is likely close to the amino group of SAM based on solved structures of the METTL3•SAH complex (PDB ID: 5IL1 and 5K7U) [53,61]. In addition, F187W stabilized the ternary complex, presumably because the larger ring of tryptophan can interact more favorably than phenylalanine with the adenosine (Figure S2) [62]. R282 is immediately adjacent to the catalytic core in the METTL16•MAT2A hp1 complex and METTL16 R282A exhibited no measurable activity, although it is not clear if it is a problem with binding to SAM, catalysis, or both (Table S2) [17].

METTL16 may promote or prevent cancer depending on cancer type (see recent review [63,64]). SAM homeostasis is partly regulated by METTL16 and the methylation status of MAT2A mRNA, whereby methylation of MAT2A hps inhibits SAM production [10,11,17,18]. Thus, METTL16 contributes to the cellular methylation potential, which is the relative abundance of SAM and SAH. Aberrant methylation patterns, whether it be hypomethylation or hypermethylation of DNA, RNA or proteins, enables cancer through various mechanisms [65]. Specifically, intracellular SAM levels modulated by METTL16 alter m^6^A and histone methylation profiles [10,57,59,60]. Examples of METTL16 affecting m^6^A profiles in colorectal cancer include the Soga1 and SSB mRNAs [51,66]. Our kinetic analysis of METTL16 cancer-associated mutants identified one mutant, METTL16 R200Q, with increased catalytic efficiency (Table 5), which is consistent with this mutant showing enhanced methylation activity in previous cell-based assays [61]. This enhanced activity would lead to decreased MAT2A levels, which presumably would reduce the cellular methylation potential by reducing intracellular SAM and lead to global hypomethylation in a cell [61]. In contrast, METTL16 cancer-associated mutants G110C and R241Dfs*2 displayed dramatically reduced methylation activity in our kinetic assay (Table 5) and their loss-of-function may play a role in causing and/or promoting cancer. METTL16 G110C can bind to RNA but not SAM (Table 5 and Table S1), suggesting that G110 may interact directly with the amino group of SAM as observed in an X-ray crystal structure of a METTL16•SAH complex (PDB ID: 2H00). We speculate that weak SAM binding may cause the METTL16 G110C mutant to remain bound to MAT2A mRNA for extended times and enable proper expression of MAT2A to increase intracellular SAM concentrations and subsequently global hypermethylation within a cell. R241Dfs*2 shows poor RNA binding, which is required for MAT2A regulation, so a decrease in the MAT2A enzyme and overall low cellular methylation potential would be expected. In addition, cancer-associated mutations, particularly extreme truncations like R241Dfs*2, may be unstable and have shorter intracellular half-lives, further exacerbating the loss of activity. Besides MAT2A, hypomethylation of U6 snRNA at A43 could result in mis-splicing events that contribute to cancer, for there is a longstanding link between aberrant splicing and cancer [20,67]. Another consideration is how METTL16 cancer-associated mutants participate in methylation-independent functions and/or alterations in other genes, such as MTAP [10]. It is possible that METTL16 cancer-associated mutations, particularly residues mutated in the C-terminal region (E408K, P460L, R552H, and T549A), could interfere with protein–protein interactions or other functions such as the autoregulatory loop involving SSB in colorectal cancer [51]. However, METTL16 mutants that have no methylation activity or even improved activity, such as the cancer-associated mutant R200Q, maintained their interactions with eukaryotic initiation factors 3a and 3b, an interaction that promotes cancer via translation of certain mRNAs [40]. Because METTL16 has such diverse cellular roles, its direct role in cancer is likely mechanistically complex.

## 5. Conclusions

Our mutational analysis has provided insights into how specific residues and domains contribute to the functional activity of METTL16 with respect to U6 snRNA. The C-terminal domain contributes more to the cooperative binding and recognition of U6 snRNA than the methyltransferase domain. The bulky side chains of the K-loop obstruct SAM binding and may potentially limit the rate of methylation. Furthermore, mutations of residues in the SAM-binding pocket, such as R82 and E133, had similar deleterious effects on U6 snRNA methylation as seen with MAT2A mRNA hp1 methylation. We also identified two phenylalanine residues adjacent to the adenosine nucleobase of SAM with strikingly different effects on catalysis: F188 contributes to the rate of methylation, while F227 supports SAM binding. Lastly, most cancer-associated mutants were able to bind and methylate U6 snRNA similar to full-length METTL16, except METTL16, G110C, and R241Dfs*2, which decreased methylation by 113-fold or more. This study, coupled with the known three-dimensional structures of METTL16 in bound and unbound states, highlights the dynamic interplay of substrate binding, catalysis, and residue motions during the catalytic cycle, which in turn impact intracellular levels of SAM. Such mechanistic information may aid in the development of METTL16-specific inhibitors and activators [68,69].

## Figures and Tables

**Table 1:** Dissociation constants of METTL16•U6 snRNA.

METTL16	*K*_D1_ (nM)	Degree of Cooperativity	Fold Weaker RNA Binding ^a^
***Full-length (FL) and truncated METTL16***			
FL (1–562)	132 ± 15	5 ± 1	-
METTL16_291 (1–291)	5200 ± 1300	1.6 ± 0.1	39
METTL16Δ1-291 (291–562)	814 ± 90	3.3 ± 0.5	6.2
***RNA-binding region (1–79)***			
K5A	126 ± 10	7 ± 1	0.95
K5A/R10A	175 ± 27	6.8 ± 0.6	1.3
K5A/R10A/R12A	231 ± 58	4 ± 2	1.8
K5A/R10A/R12A/K14A	353 ± 25	6 ± 1	2.7
K5A/R10A/R12A/K14A/K16A	293 ± 35	4.3 ± 0.2	2.2
N39A	187 ± 0.5	7 ± 1	1.4
***Rossmann fold (80–291)***			
R82A	306 ± 9	6.8 ± 0.6	2.3
F187G	330 ± 12	6.8 ± 0.4	2.5
F187W	276 ± 12	6.0 ± 0.8	2.1
R282A	307 ± 28	6.0 ± 0.3	2.3
***Arginine-rich region (382–388) in VCR1***			
R382A	294 ± 13	7 ± 1	2.2
R382A/R383A/R386A/R388A	649 ± 33	2.9 ± 0.1	4.9
ΔR382-R388	1339 ± 122	2.7 ± 0.8	10.1

^a^ Calculated as (*K*_D1_)_Mutant_/(*K*_D1_)_FL_. Colors correspond to regions of METTL16 as defined in Figure 1C.

**Table 2:** Kinetic parameters of METTL16 mutants targeting K-loop residues.

METTL16	*K*_D2_ (µM)	*k*_chem_ (min^−1^)	*k*_chem_/*K*_D2_ (µM^−1^min^−1^)	Fold Tighter SAM Binding ^a^
FL METTL16 ^b^	126 ± 6	0.56 ± 0.01	0.0044	-
Q162A	77 ± 6	0.49 ± 0.01	0.0064	1.6
K163A	23 ± 2	0.48 ± 0.01	0.021	5.5
M167A	43 ± 10	0.52 ± 0.03	0.012	2.9
K163A/M167A	14 ± 5	1.30 ± 0.07	0.093	9
Q162A/K163A/M167A	13 ± 2	1.98 ± 0.05	0.15	9.7

^a^ Calculated as a (*K*_D2_)_FL_/(*K*_D2_)_Mutant_. ^b^ Reported values were obtained from reference [14].

**Table 3:** Kinetic parameters of METTL16 mutants targeting the SAM-binding site.

METTL16	*K*_D2_ (µM)	*k*_chem_ (min^−1^)	*k*_chem_/*K*_D2_ (µM^−1^min^−1^)	Fold Weaker SAM Binding ^a^
FL METTL16 ^b^	126 ± 6	0.56 ± 0.01	0.0044	-
METTL16_291 ^b^	736 ± 94	0.42 ± 0.02	5.7 × 10^−4^	5.8
R82A	280 ± 38	0.012 ± 0.001	4.3 × 10^−5^	2.2
T111A	86 ± 9	0.45 ± 0.01	0.0052	0.7
S114A	286 ± 19	0.37 ± 0.01	0.0013	2.3
E133A	>1000	0.04 ± 0.01 ^c^	<4 × 10^−5^	>7.9
F188A	383 ± 97	0.018 ± 0.002	4.7 × 10^−5^	3.0
T216A	42 ± 11	0.29 ± 0.01	0.0069	0.3
F227A	>1000	0.36 ± 0.13	<3.6 × 10^−5^	>7.9
R230A	70 ± 5	0.54 ± 0.01	0.0077	0.6

^a^ Calculated as a (*K*_D2_)_Mutant_/(*K*_D2_)_FL_. ^b^ Reported values were obtained from reference [14]. ^c^ Highest *k*_obs_ value observed at 1 mM SAM; Equation (3) fitting did not converge.

**Table 4:** Kinetic parameters of METTL16 mutants targeting the catalytic core.

METTL16	*K*_D2_ (µM)	*k*_chem_ (min^−1^)	*k*_chem_/*K*_D2_ (µM^−1^min^−1^)	Relative Catalytic Efficiency ^a^
FL METTL16 ^b^	126 ± 6	0.56 ± 0.01	0.0044	--
N184A	--	No measurable activity	--	--
N184D	--	No measurable activity	--	--
N184D/F187W	--	No measurable activity	--	--
P185A/P186A	--	No measurable activity	--	--
F187G	227 ± 36	0.0034 ± 0.0002	1.5 × 10^−5^	↓ 293
F187W	38 ± 10	0.44 ± 0.02	0.012	↑ 2.7

^a^ Calculated as a (*k*_chem_/*K*_D2_)_FL_/(*k*_chem_/*K*_D2_)_Mutant_ for ↓ and as (*k*_chem_/*K*_D2_)_Mutant_/(*k*_chem_/*K*_D2_)_FL_ for ↑. ^b^ Reported values were obtained from reference [14].

**Table 5:** Kinetic parameters and dissociation constants of METTL16 cancer-associated mutants.

METTL16 Cancer-Associated Mutation (Cancer Type)	*K*_D1_ (nM)	*K*_D2_ (µM)	*k*_chem_ (min^−1^)	*k*_chem_/*K*_D2_ (µM^−1^min^−1^)	Relative Catalytic Efficiency ^a^
	Degree of Cooperativity				
FL METTL16 ^b^	132 ± 15	126 ± 6	0.56 ± 0.01	0.0044	-
	5 ± 1				
METTL16_291 (1–291)	5200 ± 1300	736 ± 94	0.42 ± 0.02	5.7 × 10^−4^	↓ 7.7
	1.6 ± 0.1				
***Rossmann fold***					
G110C (Intestinal)	179 ± 19	>1000	0.005 ± 0.003 ^c^	<5 × 10^−6^	↓ >880
	14 ± 10				
R200Q (Intestinal)	160 ± 6	66 ± 9	0.41 ± 0.01	0.0062	↑ 1.4
	9.8 ± 0.3				
R241Dfs*2 (Colorectal)	5960 ± 420	--	No measurable activity	--	--
	4.9 ± 0.2				
***VCR1***					
E408K (Esophageal)	181 ± 2	134 ± 21	0.48 ± 0.02	0.0036	↓ 1.2
	5.6 ± 0.7				
***Disordered region***					
P460L (Liver)	131 ± 4	149 ± 21	0.53 ± 0.02	0.0036	↓ 1.2
	6.4 ± 0.8				
***VCR2***					
T549A (Central Nervous System)	148 ± 2	120 ± 4	0.44 ± 0.01	0.0037	↓ 1.2
	9 ± 2				
R552H (Stomach)	137 ± 9	125 ± 10	0.50 ± 0.01	0.0040	↓ 1.1
	7.1 ± 0.4				

^a^ Calculated as a (*k*_chem_/*K*_D2_)_FL_/(*k*_chem_/*K*_D2_)_Mutant_ for ↓ and as (*k*_chem_/*K*_D2_)_Mutant_/(*k*_chem_/*K*_D2_)_FL_ for ↑. ^b^ Reported values were obtained from reference [14]. ^c^ Highest *k*_obs_ value observed at 1 mM SAM. Colors correspond to regions of METTL16 as defined in Figure 1C.

## Data Availability

Analyzed data are included in the article/Supplementary Material. Further inquiries can be directed to the corresponding author.

## Supplementary Materials

The following supporting information can be downloaded at https://www.mdpi.com/article/10.3390/biology14091145/s1, Figure S1: CD spectra of METTL16 mutants with decreased activity; Figure S2: Structural basis of F187 interacting with RNA; Figure S3: Location of T216 in METTL16•RNA and METTL16•SAH complexes; Figure S4: List of somatic METTL16 cancer-associated mutations examined and their locations; Figure S5: Uncropped gel images for Figure 2C; Table S1: *K*_D1_ values for RNA binding activity of METTL16 mutants targeting SAM-binding pocket, _184_NPPF_187_ catalytic core, and K-loop; Table S2: Single-turnover kinetic parameters of METTL16 mutants targeting RNA-binding site; Supplementary File S1: Analyzed data. References [14,25] are cited in the Supplementary Materials.

## Abbreviations

The following abbreviations are used in this manuscript:

CD	Circular dichroism
EMSA	Electrophoretic mobility shift assay
FL	Full-length
hp	Hairpin
hps	Hairpins
*k*_chem_	Rate constant for methylation
*K*_D_	Equilibrium dissociation constant
m6A	*N*^6^-methyladenosine
MAT2A	Methionine adenosyltransferase 2A
METTL	Methyltransferase-like protein
MTAP	Methylthioadenosine phosphorylase
PDB ID	Protein Data Bank identifier
RNP	Ribonucleoprotein
SAM	*S*-adenosylmethionine
SAH	*S*-adenosylhomocysteine
snRNA	Small nuclear RNA
Soga	Suppressor of glucose autophagy
SSB	Small RNA binding exonuclease protection factor La
TLC	Thin layer chromatography
VCR	Vertebrate conserved region
