# Supplementary material for: Mutational Analysis Reveals Functional Roles of METTL16 Domains and Residues
Source: Biology (Basel). 2025 Aug 29;14(9):1145. doi: 10.3390/biology14091145 (PMC12467241; doi:10.3390/biology14091145)
Supplement: Supplementary file 1 [file biology-14-01145-s001.zip › biology-3823960-Supplementary Materials-proof-done/biology-3823960-Supplementary Figures and Tables-proofed.pdf]

biology

SUPPLEMENTARY MATERIALS

Mutational Analysis Reveals Functional Roles of METTL16 Domains and Residues

Kurtis Breger [1], Ian P. Schowe [1], Noah A. Springer [1], Nathan J. O'Leary [1], Agnieszka Ruszkowska [1,#], Carlos Resende [1] and Jessica A. Brown [1,*]

[1] Department of Chemistry and Biochemistry, University of Notre Dame, Notre Dame, IN 46556 USA
[#] Current address: Department of Structural Chemistry and Biology of Nucleic Acids, Institute of Bioorganic Chemistry, Polish Academy of Sciences, Poznan 61-704, Poland
* Correspondence: jbrown33@nd.edu

Supplementary Figures

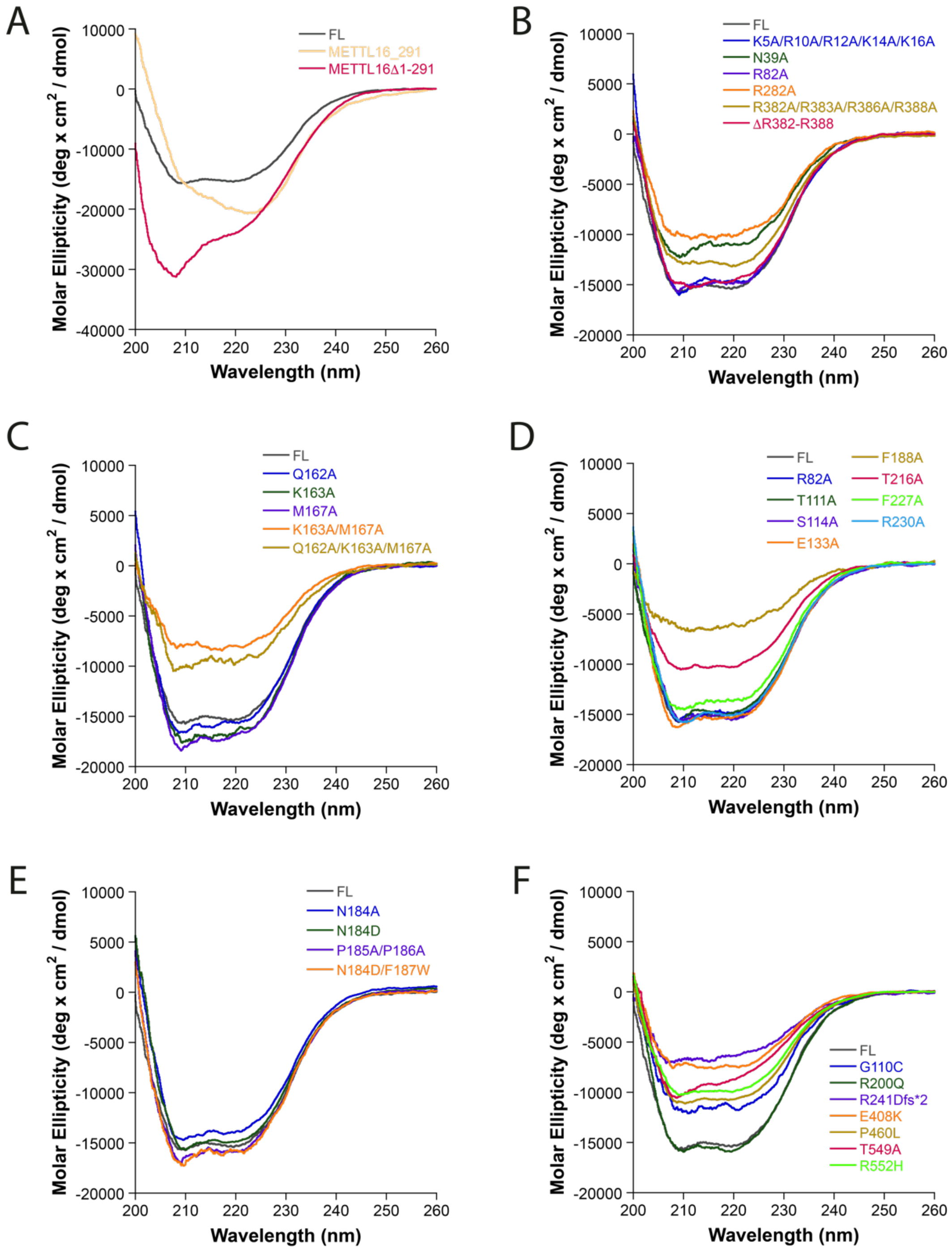

Figure S1. CD Spectra of METTL16 mutants with decreased activity. CD spectra for (A) N- and C-terminal domain truncations, (B) RNA-binding, (C) K-loop, (D) SAM-binding pocket, (E) $_{184}$NPPF$_{187}$ catalytic core, and (F) somatic cancer residues. For all plots, full-length METTL16 is displayed as a gray line.

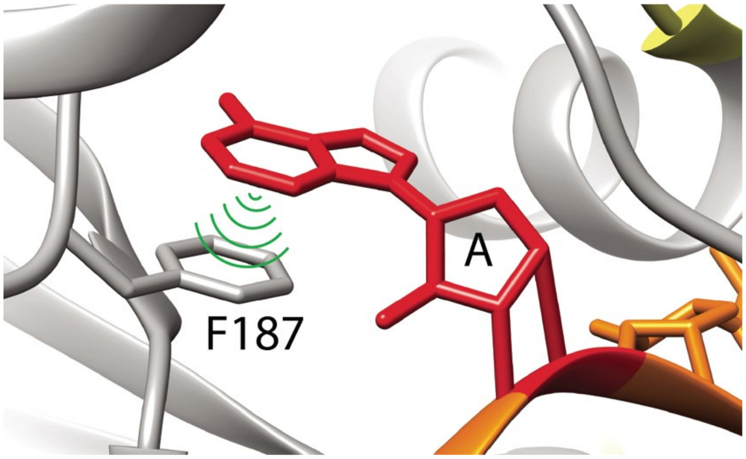

Figure S2. Structural basis of F187 interacting with RNA. The METTL16•MAT2A hp1 crystal structure (PDB ID: 6DU4) indicates F187 can pi-stack (green arcs) with methyl acceptor adenosine (red) in MAT2A hp1 (orange).

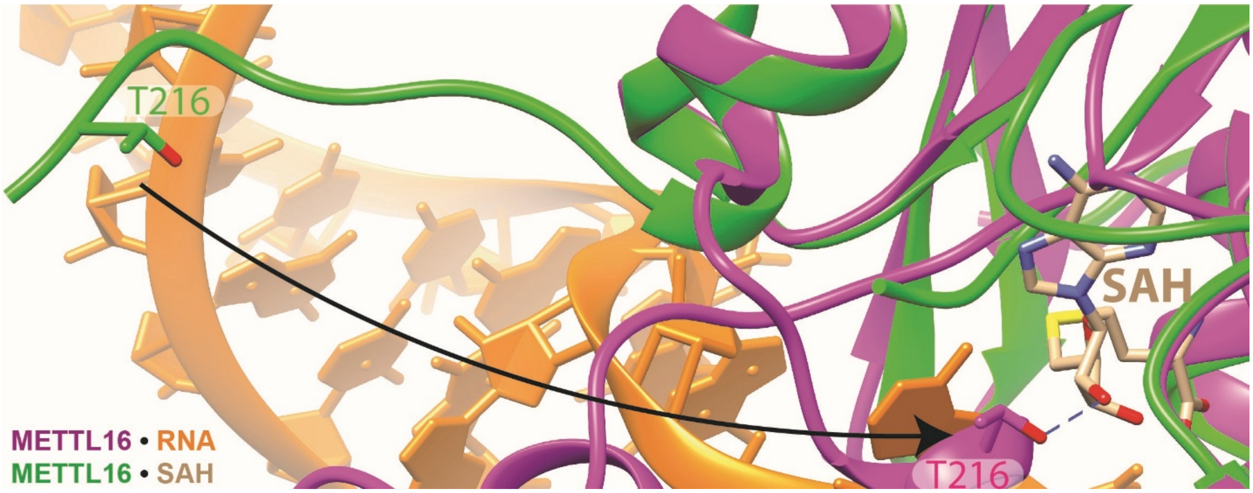

Figure S3. Location of T216 in METTL16•RNA and METTL16•SAH complexes. T216 resides in drastically different locations in complexes of the METTL16•MAT2A hp1 RNA (PDB ID: 6DU4), where METTL16 and RNA are shown in magenta and orange, respectively, versus METTL16•SAH (PDB ID: 6B92), where METTL16 and SAH are shown in green and tan, respectively. The T216 residue in the METTL16•RNA crystal structure places the threonine side chain in proximity of the O2′ ribose of SAH, suggesting a potential hydrogen bond between the two moieties.

biology

MDPI

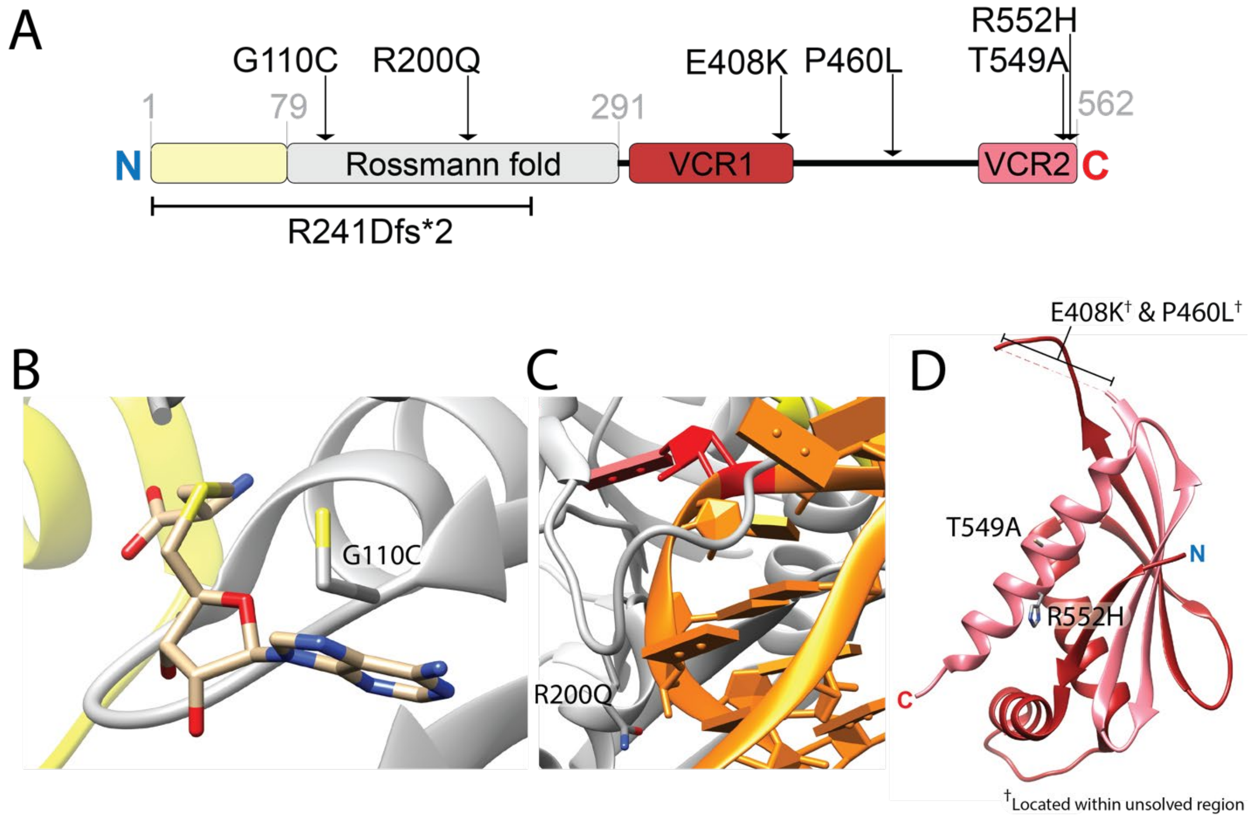

Figure S4. List of somatic METTL16 cancer-associated mutations examined and their locations. (A) Point mutations include G110C, R200Q, E408K, P460L, T549A, and R552H. The frameshift mutant R241Dfs*2 results in a truncated form of METTL16. (B) The G110C mutation introduces a cysteine within the *GXG* motif, which outlines the SAM/SAH-binding site (PDB ID: 6B92). (C) The R200Q mutation resides near the transition of unstructured-to-structured RNA of the MAT2A hp1 (PDB ID: 6DU4). (D) E408K and P460L cannot be displayed because they were not present in crystal structure of the VCRs. The T549A and R552H mutations are located within VCR2 near the end of METTL16 (PDB ID: 6M1U). For panels B-D, point mutants were modeled using UCSF Chimera [25].

biology

MDPI

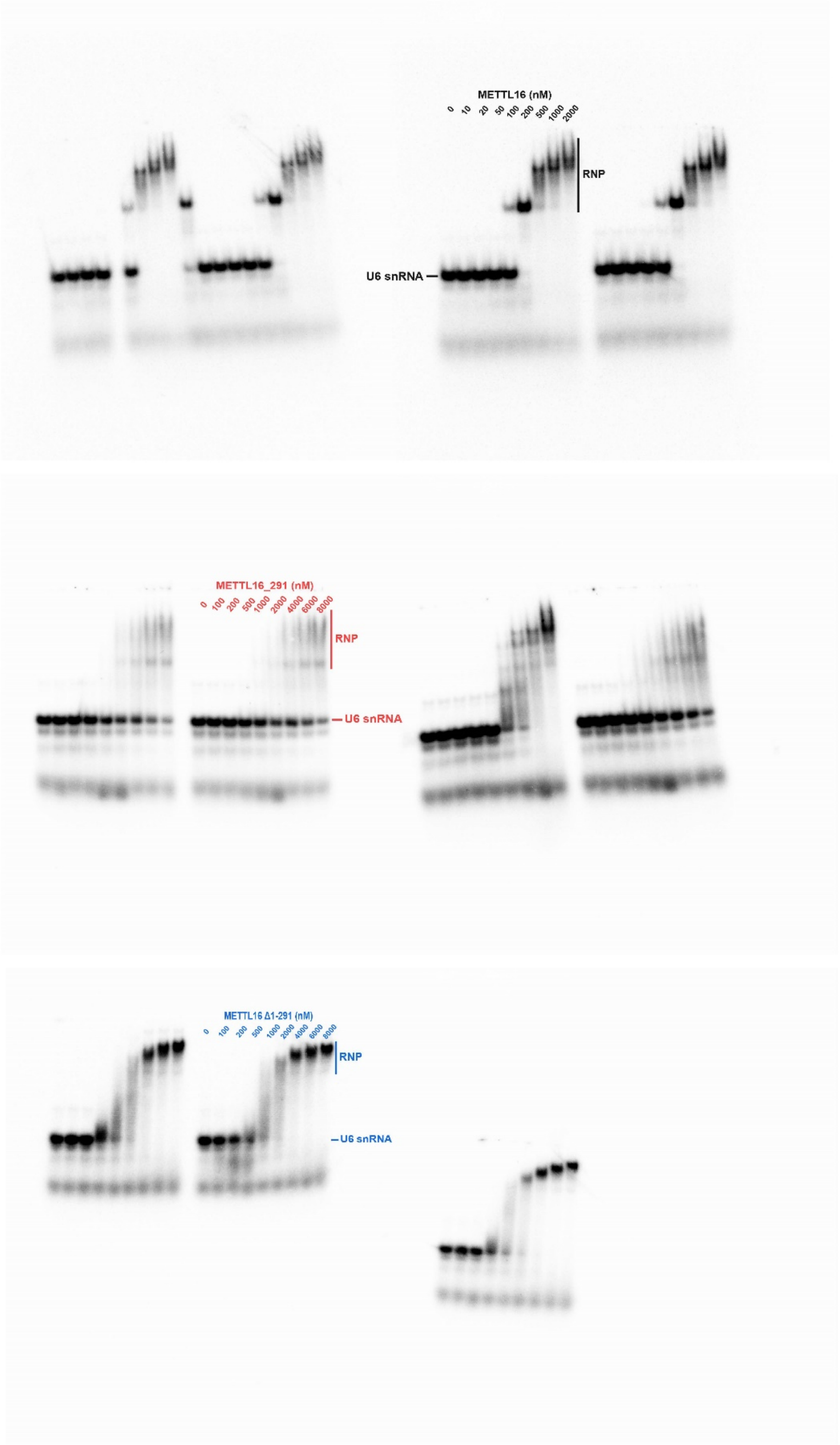

Figure S5. Uncropped gel images for Figure 2C.

biology

MDPI

Supplementary Tables

Table S1. K_{D1} values for RNA binding activity of METTL16 mutants targeting SAM-binding pocket, $_{184}$NPPF$_{187}$ catalytic core, and K-loop.

**METTL16 Mutant**	**K_{D1} (nM)**	**Degree of Cooperativity**	**Fold Weaker RNA Binding[a]**
FL (1-562)	132 ± 15	5 ± 1	-
K-loop Residues			
Q162A	150 ± 2	7.6 ± 0.6	1.1
K163A	166 ± 20	14 ± 8	1.3
M167A	155 ± 5	7.1 ± 0.3	1.2
K163A/M167A	191 ± 3	10 ± 2	1.4
Q162A/K163A/M167A	268 ± 67	5.7 ± 0.8	2.0
SAM-Binding Residues			
T111A	134 ± 13	5.4 ± 0.9	1.0
S114A	136 ± 24	5.8 ± 0.7	1.0
E133A	139 ± 4	11 ± 1	1.1
F188A	301 ± 41	5.9 ± 0.7	2.3
T216A	128 ± 2	9.4 ± 0.3	0.97
F227A	163 ± 13	7 ± 1	1.2
R230A	149 ± 5	7.6 ± 0.3	1.1
NPPF Catalytic Core			
N184A	202 ± 2	6.9 ± 0.5	1.5
N184D	200 ± 25	7 ± 1	1.5
N184D/F187W	239 ± 55	6 ± 1	1.8
P185A/P186A	157 ± 4	9.0 ± 0.7	1.2
[a] Calculated as $(K_{D1})_{Mutant}$ / $(K_{D1})_{FL}$.			

Table S2. Single-turnover kinetic parameters of METTL16 mutants targeting RNA-binding site.

**METTL16 Mutant**	K_{D2} (μM)	k_{chem} (min^{-1})	k_{chem}/K_{D2} ($\mu M^{-1} min^{-1}$)	**Relative Catalytic Efficiency**[a]
Full-length and Truncated METTL16				
FL (1-562) [b]	126 ± 6	0.56 ± 0.01	0.0044	--
METTL16_291 (1-291) [b]	736 ± 94	0.42 ± 0.02	5.7×10^{-4}	↓ 7.7
METTL16Δ1-291 (292-562)	N/A	N/A	N/A	N/A
RNA-Binding Region (1-79)				
K5A	92 ± 8	0.44 ± 0.01	0.0047	↑ 1.1
K5A/R10A	61 ± 11	0.42 ± 0.02	0.0069	↑ 1.6
K5A/R10A/R12A	108 ± 14	0.42 ± 0.02	0.0039	↓ 1.1
K5A/R10A/R12A/K14A	97 ± 4	0.379 ± 0.004	0.0039	↓ 1.1
K5A/R10A/R12A/K14A/K16A	71 ± 9	0.35 ± 0.01	0.0049	↑ 1.1
N39A	131 ± 50	0.52 ± 0.06	0.0039	↓ 1.1
Rossmann Fold (80-291)				
R82A	280 ± 38	0.012 ± 0.001	4.3×10^{-5}	↓ 102
F187G	227 ± 36	0.0034 ± 0.0002	1.5×10^{-5}	↓ 293
F187W	38 ± 10	0.44 ± 0.02	0.012	↑ 2.7
R282A	--	No measurable activity	--	--
Arginine-rich Region (382-388) in VCR1				
R382A	156 ± 34	0.558 ± 0.04	0.0036	↓ 1.2
R382A/R383A/R386A/R388A	208 ± 9	0.516 ± 0.008	0.0025	↓ 1.8
ΔR382-R388	272 ± 16	0.47 ± 0.01	0.0017	↓ 2.6

biology

MDPI

[a] Calculated as $(k_{chem}/K_{D2})_{Mutant}$ / $(k_{chem}/K_{D2})_{FL}$ for ↑ and as $(k_{chem}/K_{D2})_{FL}$ / $(k_{chem}/K_{D2})_{Mutant}$ for ↓.
[b] Reported values were obtained from reference [14].
